# The Efficacy of Ketogenic Diet for Specific Genetic Mutation in Developmental and Epileptic Encephalopathy

**DOI:** 10.3389/fneur.2018.00530

**Published:** 2018-07-16

**Authors:** Ara Ko, Da E. Jung, Se H. Kim, Hoon-Chul Kang, Joon S. Lee, Seung T. Lee, Jong R. Choi, Heung D. Kim

**Affiliations:** ^1^Division of Pediatric Neurology, Department of Pediatrics, Epilepsy Research Institute, Severance Children's Hospital, Yonsei University College of Medicine, Seoul, South Korea; ^2^Department of Pediatrics, Ajou University School of Medicine, Suwon, South Korea; ^3^Department of Laboratory Medicine, Severance Hospital, Yonsei University College of Medicine, Seoul, South Korea

**Keywords:** developmental and epileptic encephalopathy, ketogenic diet, next-generation sequencing, mutation, precision medicine

## Abstract

**Objectives:** Pathogenic mutations in developmental and epileptic encephalopathy (DEE) are increasingly being discovered. However, little has been known about effective targeted treatments for this rare disorder. Here, we assessed the efficacy of ketogenic diet (KD) according to the genes responsible for DEE.

**Methods:** We retrospectively evaluated the data from 333 patients who underwent a targeted next-generation sequencing panel for DEE, 155 of whom had tried KD. Patients showing ≥90% seizure reduction from baseline were considered responders. The KD efficacy was examined at 3, 6, and 12 months after initiation. Patients were divided into those with an identified pathogenic mutation (*n* = 73) and those without (*n* = 82). The KD efficacy in patients with each identified pathogenic mutation was compared with that in patients without identified genetic mutations.

**Results:** The responder rate to KD in the patients with identified pathogenic mutations (*n* = 73) was 52.1, 49.3, and 43.8% at 3, 6, and 12 months after initiation, respectively. Patients with mutations in *SCN1A* (*n* = 18, responder rate = 77.8%, *p* = 0.001), *KCNQ2* (*n* = 6, responder rate = 83.3%, *p* = 0.022), *STXBP1* (*n* = 4, responder rate = 100.0%, *p* = 0.015), and *SCN2A* (*n* = 3, responder rate = 100.0%, *p* = 0.041) showed significantly better responses to KD than patients without identified genetic mutations. Patients with *CDKL5* encephalopathy (*n* = 10, responder rate = 0.0%, *p* = 0.031) showed significantly less-favorable responses to KD.

**Conclusions:** The responder rate to KD remained consistent after KD in DEE patients with specific pathogenic mutations. KD is effective in patients with DEE with genetic etiology, especially in patients with *SCN1A, KCNQ2, STXBP1*, and *SCN2A* mutations, but is less effective in patients with *CDKL5* mutations. Therefore, identifying the causative gene can help predict the efficacy of KD in patients with DEE.

## Introduction

Epileptic encephalopathy refers to a group of epileptic disorders that epileptic activities *per se* cause adverse impacts to the patient's development (1). Developmental and epileptic encephalopathy (DEE) is a recently introduced concept with the advances in genetic diagnosis, as genetic etiologies can cause developmental delay irrespective of epileptic activities (2). The causes of DEE can be various including structural and metabolic etiologies. Moreover, owing to advances in genetic testing technologies, such as next-generation sequencing (NGS), research has revealed that diverse genetic mutations, especially *de novo* monogenic mutations, constitute a significant portion of the etiologies of DEE (3). The discovery of causative genes for DEE has amplified the efforts to improve our understanding of the pathophysiology of each genetic mutation, with the ultimate objective of precision medicine. For example, ketogenic diet (KD), mechanistic target of rapamycin (mTOR)-inhibitors, and retigabine are effective targeted treatments for glucose transporter type 1 deficiency, mTORopathies, and *KCNQ2* encephalopathy, respectively (4–6).

The KD is an effective therapeutic option for various DEE syndromes (7–10), and may be an alternative to pharmacologic therapies. However, the effects of KD vary among patients and implementing KD even as a short-term trial is a great challenge for both patients and caregivers, owing to the restrictive diet regimen and potential side effects (11).

Therefore, identifying those patients who are the most likely to respond to KD would be beneficial for determining with whom and when KD should be initiated. In the present study, we aimed to establish whether the effects of KD differ according to the type of genetic mutation, as confirmed by the targeted NGS gene panel for DEE.

## Materials and methods

### Patients

We retrospectively evaluated the data from patients who were diagnosed with DEE at Severance Children's Hospital, Seoul, South Korea. Inclusion criteria were as follows: (1) patients with epilepsy and cognitive and behavioral impairments who were diagnosed with DEE; (2) patients whose seizures or developmental delays were noticed before the age of 3 years; (3) patients who underwent a targeted NGS gene panel for DEE between January 2016 and March 2017; (4) patients who failed to achieve seizure freedom with adequate trials of two or more antiepileptic drugs (AEDs); and (5) patients who started KD therapy between January 2006 and June 2016. Exclusion criteria were as follows: (1) patients with proven etiologies other than genetic etiology, such as structural, infectious, or immune encephalopathies; and (2) patients who underwent KD therapy for cognitive benefits rather than seizure control, owing to difficulties in assessing the efficacy of KD by seizure frequency.

This study was approved by the Institutional Review Board of Severance Hospital (IRB No. 4-2017-0699) and written informed consent was waived.

### Assessment of outcomes

The seizure frequencies before KD (baseline) and at 3, 6, and 12 months after KD were obtained. The seizure frequency 2 months before KD initiation was defined as the “baseline.” When assessing the seizure frequency, the number of seizures that had occurred during the previous 2 months was counted. Patients were considered KD responders if they showed ≥90% seizure reduction from the baseline seizure frequency, while patients who showed < 90% seizure reduction were considered non-responders. The long-term treatment response to KD was defined as a reduction in seizure frequency at both 6 and 12 months compared to baseline.

The variables we evaluated with respect to their possible predictive value for a responder were as follows: age at seizure onset, sex, number of AEDs being taken at the time of KD initiation, lead time from seizure onset to KD initiation, the total duration of KD implementation, epilepsy syndrome, and pathogenic mutation. Regarding the epilepsy syndrome, the syndromes of each patient at the time of KD implementation were selected. To classify the epilepsy syndrome, we used the classifications outlined by the International League Against Epilepsy in their revised terminology and concepts for organization of seizures and epilepsies (1).

### Targeted NGS panel for DEE

The targeted NGS panel for DEE at Severance Children's Hospital comprises 172 genes that are known to be related to DEE (Table S1). Using whole-blood samples, genomic DNA was extracted from leukocytes with the QIAamp Blood DNA mini kit (Qiagen, Hilden, Germany). After processing, pooled libraries were sequenced using a MiSeq sequencer (Illumina, San Diego, CA, USA) and the MiSeq Reagent Kit v2 (300 cycles). The interpretation of variants followed the 5-tier classification system recommended by the American College of Medical Genetics and Genomics and the Association for Molecular Pathology (12). Variants considered pathogenic or likely pathogenic according to the American College of Medical Genetics and Genomics and the Association for Molecular Pathology classification system were selected as causative mutations for epileptic encephalopathies (12).

### KD

Patients were instructed to follow either a classic 4:1 or 3:1 KD or modified Atkins diet by the attending pediatric epileptologists (13). Patients immediately began the diet regimen without an initial fasting period, and calories were restricted to 75% of the recommended daily intake. Screening and follow-up examinations were performed according to the protocol reported by Kang et al. (11), and included measurements of serum β-hydroxybutyrate and urine ketone bodies to assess ketosis and adjust the ratios if required.

### Statistical analysis

Values are expressed as the median with the interquartile range (IQR) or as the number and percentage. Comparisons between two groups were performed using chi-squared tests or Fisher's exact tests for categorical data or using Mann-Whitney *U*-tests for non-parametric and continuous data. Statistical significance was set at *p* < 0.05. The Statistical Package for the Social Sciences software (version 23.0; SPSS Inc., Chicago, IL, USA) was used for all analyses.

## Results

### Patients and clinical characteristics

**Figure 1:**
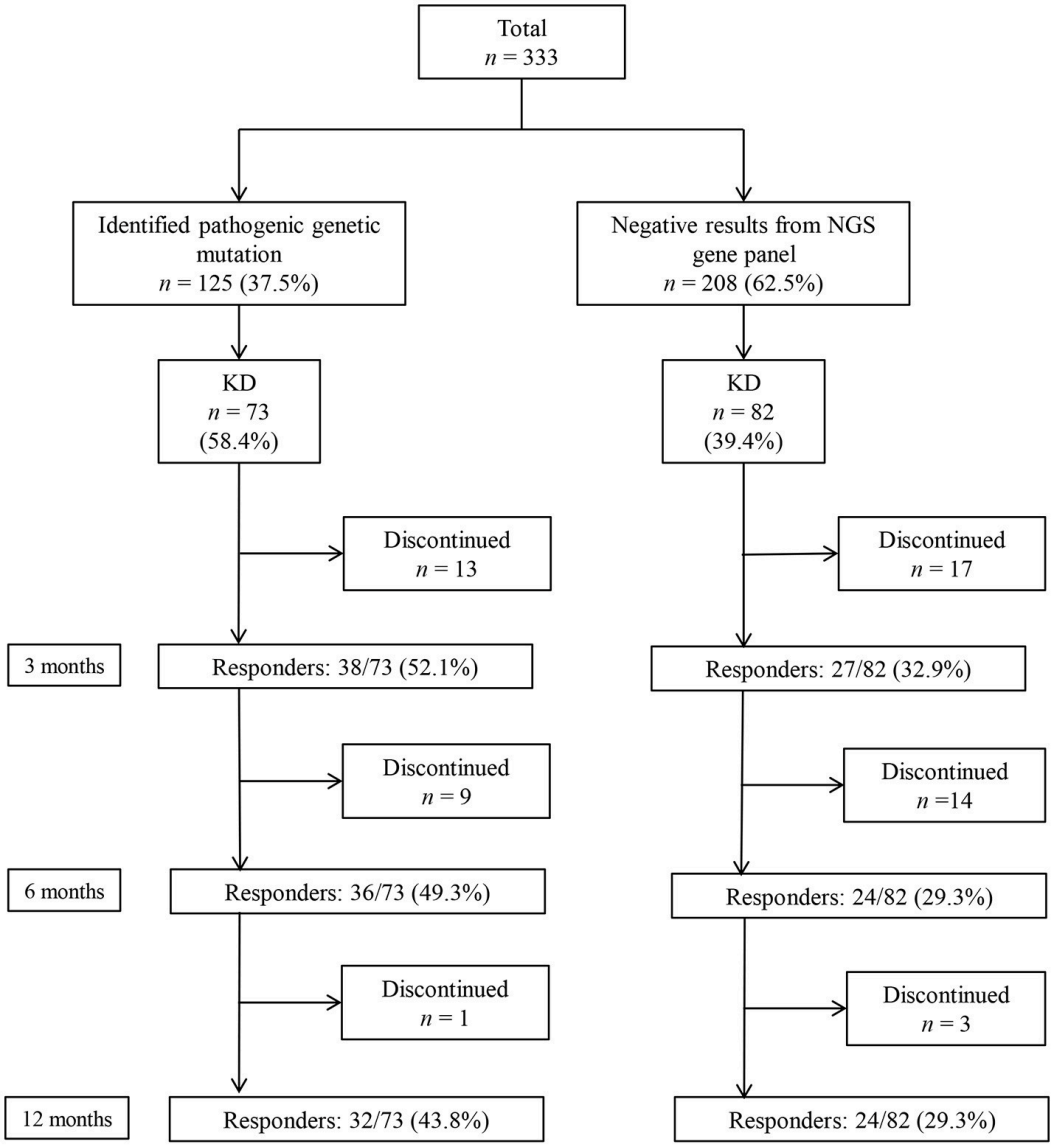
Responses to a ketogenic diet (KD) after 3, 6, and 12 months in patients with and without identified genetic mutations, as determined by a targeted next-generation sequencing (NGS) gene panel for developmental and epileptic encephalopathy.

A total of 333 patients (median age 7 [IQR: 3–18] months, 189 [56.8%] boys) who underwent a targeted NGS gene panel for DEE were included in this retrospective cohort study. Among the 333 patients, 125 (37.5%) patients received a genetic diagnosis. Of the 125 patients with a monogenic mutation, 73 had tried KD, while 82 of the 208 patients without an identified genetic mutation had tried KD (Figure 1). In total, 155 patients (73 with an identified genetic mutation and 82 without) were subjected to the analysis to determine the KD efficacy. Of the 73 patients with identified genetic mutations, 38 (52.1%) patients responded to KD at 3 months, 36 (49.3%) responded at 6 months, and 32 (43.8%) responded at 12 months (Figure 1). Patients were on KD for a median duration of 19 months (IQR: 11.0–42.5, range: 1–143 months). In the group of 73 patients with an identified genetic mutation, 50 (68.5%) patients were still on KD after 12 months. Thirteen patients discontinued KD due to inefficacy within 3 months, nine patients discontinued KD between 3 and 6 months (six due to inefficacy, one to aspiration pneumonia, one to recurrent acidosis, and one due to noncompliance), and one patient discontinued KD between 6 and 12 months due to noncompliance. Of the 82 patients without an identified genetic mutation, 27 (32.9%) patients responded to KD at 3 months, and 24 (29.3%) responded at both 6 and 12 months. Among the patients without an identified genetic mutation, 48 (58.5%) patients remained on KD after 12 months. Seventeen patients discontinued the diet within 3 months (eleven due to inefficacy, one to recurrent acidosis, one to recurrent hypoglycemia, one to pancreatitis, one to intolerable diarrhea, and two due to noncompliance). Fourteen patients discontinued the diet between 3 and 6 months (eight due to inefficacy, three to noncompliance, two to aspiration pneumonia, and one due to metabolic encephalopathy), and three patients discontinued the diet between 6 and 12 months (one due to inefficacy and two to noncompliance).

The clinical characteristics of the 155 patients who underwent the NGS gene panel and tried KD are summarized in Table 1. The median age at seizure onset was 6.0 (IQR: 3.0–15.0) months, and 65.2% of the patients were boys. The median seizure frequency was 7.0 (IQR: 2.0–20.0) seizures per day, while the median number of AEDs patients were taking at the time of KD initiation was 3 (IQR: 3–4). The median lead time from seizure onset to KD initiation was 11.0 (IQR: 5.0–30.5) months. Initially, 24 (15.1%) patients received the 4:1 KD, 65 (41.9%) received the 3:1 KD and 66 (42.6%) received the modified Atkins diet. Regarding the syndromic diagnosis at the time of KD implementation, West syndrome was the most frequent (67/155 patients, 43.2%), followed by Lennox-Gastaut syndrome (31/155 patients, 20.0%), and Dravet syndrome (18/155 patients, 11.6%), among others. None of the patients underwent epilepsy surgery or the implantation of a vagus nerve stimulation device during the first year of KD (Table 1).

**Table 1:** Demographics of patients and comparison between responders and non-responders at 3 months after KD Initiation.

**Clinical variables**	**Total (*n* = 155)**	Responders^*^ (*n* = 65)	**Non-responders (*n* = 90)**	***p***
Age at seizure onset, months	6.0 (3.0–15.0)	6.0 (3.5–11.5)	6.0 (3.0–30.8)	0.291
Sex, male	101 (65.2%)	38 (58.5%)	62 (68.9%)	0.252
Baseline seizure frequency before KD, number per day	7.0 (2.0–20.0)	6.0 (1.0–25.0)	10.0 (3.0–20.0)	0.435
Number of AEDs before KD	3 (3–4)	3 (2–4)	3 (3-4)	0.093
Lead time from seizure onset to KD, months	11.0 (5.0–30.0)	13.0 (6.0–42.5)	9.5 (5.0–24.0)	0.207
KD ratio				0.113
4:1	24	8 (33.3%)	16 (66.7%)	
3:1	65	23 (35.4%)	42 (64.6%)	
MAD	66	34 (51.5%)	32 (48.5%)	
Syndromic diagnosis				0.005
EMAS	5	1 (20.0%)	4 (80.0%)	
Dravet syndrome	18	14 (77.8%)	4 (22.2%)	
Unspecified focal epilepsy	8	2 (25.0%)	6 (75.0%)	
Unspecified generalized epilepsy	2	1 (50.0%)	1 (50.0%)	
West syndrome	67	24 (35.8%)	43 (64.2%)	
Lennox-Gastaut syndrome	31	9 (29.0%)	22 (71.0%)	
EIMFS	6	2 (33.3%)	4 (66.7%)	
Landau-Kleffner syndrome	1	1 (100.0%)	0 (0.0%)	
Ohtahara syndrome	17	11 (64.7%)	6 (35.3%)	
Identified pathogenic variant	73 (47.1%)	38 (58.5%)	35 (38.9%)	0.016

*Data are presented as the median (interquartile range) or as the number (percent)*.

* *Responders to KD represent patients who showed ≥ 90% seizure reduction from baseline*.

*KD, ketogenic diet; AED, antiepileptic drug; MAD, modified Atkins diet; EMAS, epilepsy with myoclonic atonic seizures; EIMFS, epilepsy of infancy with migrating focal seizures*.

### Clinical characteristics of KD responders vs. non-responders among patients with DEE

Among the 155 patients, 65 (41.9%) patients showed ≥90% seizure reduction from baseline at 3 months after KD initiation and these patients were considered responders. The other 90 (58.1%) patients who showed < 90% seizure reduction were considered non-responders. No statistically significant differences in the clinical variables including the age at seizure onset, sex, baseline seizure frequency, number of AEDs being taken at the time of KD implementation, lead time from seizure onset to KD, and KD ratio, were identified between responders and non-responders (Table 1). However, a significant difference was found between KD responders and non-responders regarding the syndromic diagnosis (*p* = 0.005), and significantly more patients with identified pathogenic mutations belonged to the responder group than to the non-responder group (*p* = 0.016), i.e., patients with an identified genetic mutation responded better to KD.

### Clinical variables of patients with identified mutations vs. without identified mutations by gene panel analysis

Among the 155 patients, gene panel analysis for DEE showed identified mutations in 73 (47.1%) patients. Clinical variables such as age at seizure onset, baseline seizure frequency before KD, number of AEDs taken before KD, lead time from seizure onset to KD, and KD ratio were not significantly different between patients with identified mutations and patients without identified mutations (Table 2). Proportion of girls was significantly higher in patients with identified mutations. Also, proportions of patients with identified mutations were significantly different among syndromes.

**Table 2:** Demographics of patients and comparison between patient with identified mutations and without identified mutations by gene panel analysis at 3 months after KD Initiation.

**Clinical variables**	**Total (*n* = 155)**	**Identified mutations (*n* = 73)**	**Without identified mutations (*n* = 82)**	***p***
Age at seizure onset, months	6.0 (3.0–15.0)	5.0 (2.0–10.0)	7.0 (3.0–17.3)	0.573
Sex, male	101 (65.2%)	40 (54.8%)	61 (74.4%)	0.012
Baseline seizure frequency before KD, number per day	7.0 (2.0–20.0)	6.0 (1.8–200)	7.0 (2.0–35.0)	0.675
Number of AEDs before KD	3 (3–4)	3 (2–4)	3 (3-4)	0.254
Lead time from seizure onset to KD, months	11.0 (5.0–30.0)	13.0 (5.0–37.0)	10.0 (5.0–24.0)	0.291
KD ratio				0.249
4:1	24	9 (37.5%)	15 (62.5%)	
3:1	65	28 (43.1%)	37 (56.9%)	
MAD	66	36 (54.5%)	30 (45.5%)	
Syndromic diagnosis				<0.001
EMAS	5	2 (40.0%)	3 (60.0%)	
Dravet syndrome	18	18 (100.0%)	0 (0.0%)	
Unspecified focal epilepsy	8	1 (12.5%)	7 (87.5%)	
Unspecified generalized epilepsy	2	2 (100.0%)	0 (0.0%)	
West syndrome	67	20 (29.9%)	47 (70.1%)	
Lennox-Gastaut syndrome	31	12 (38.7%)	19 (61.3%)	
EIMFS	6	3 (50.0%)	3 (50.0%)	
Landau-Kleffner syndrome	1	0 (0.0%)	1 (100.0%)	
Ohtahara syndrome	17	15 (88.2%)	2 (11.8%)	

*Data are presented as the median (interquartile range) or as the number (percent)*.

*KD, ketogenic diet; AED, antiepileptic drug; MAD, modified Atkins diet; EMAS, epilepsy with myoclonic atonic seizures; EIMFS, epilepsy of infancy with migrating focal seizures*.

### Causative monogenic mutations

Twenty-five different causative monogenic mutations were found in 73 patients (Table 2). The most common mutations were in the *SCN1A* gene (*n* = 18 [24.7%]; 10 boys and 8 girls; 11 missense mutations, four nonsense mutations, one splicing-site mutation, one exon 20 deletion, and one whole-gene deletion). Followed by the *CDKL5* (*n* = 10 [13.7%]; 2 boys and 8 girls; four missense mutations, three nonsense mutations, and three splicing-site mutations), *KCNQ2* (*n* = 6 [8.2%]; 4 boys and 2 girls; all missense mutations), and *STXBP1* (*n* = 4 [5.5%]; 2 boys and 2 girls; three missense mutations and one nonsense mutation) genes. Three patients (4.1%) had pathogenic mutations in *CHD2* (2 boys and 1 girl; two nonsense mutations and one exon 5 deletion), *KCNT1* (2 boys and 1 girl; all missense mutations), *MECP2* (3 boys; all duplications), *SCN2A* (1 boy and 2 girls; all missense mutations), and *SCN8A* (2 boys and 1 girl; all missense mutations), respectively. Other causative monogenic mutations are shown in Table 3.

**Table 3:** Responder rates to ketogenic diet according to the identified pathogenic gene, with the *P* value for the comparison with the responder rate of patients without identified genetic mutations.

**Pathogenic gene (*n* = 73)**	**At 3 months**		**At 6 months**		**At 12 months**	
	**Responders**	***p***	**Responders**	***p***	**Responders**	***p***
*ARX* (1)	1/1 (100.0%)	0.337	1/1 (100.0%)	0.301	1/1 (100.0%)	0.301
*CACNA1A* (1)	1/1 (100.0%)	0.337	0/1 (0.0%)	1.000	0/1 (0.0%)	1.000
*CDKL5* (10)	0/10 (0.0%)	0.031	0/10 (0.0%)	0.058	0/10 (10.0%)	0.058
*CHD2* (3)	1/3 (33.3%)	1.000	0/3 (0.0%)	0.555	0/3 (0.0%)	0.555
*CLCN4* (1)	0/1 (0.0%)	1.000	0/1 (0.0%)	1.000	0/1 (0.0%)	1.000
*COL4A1* (2)	1/2 (50.0%)	1.000	1/2 (50.0%)	0.509	1/2 (50.0%)	0.509
*EEF1A2* (2)	0/2 (0.0%)	1.000	0/2 (0.0%)	1.000	0/2 (0.0%)	1.000
*GNAO1* (1)	0/1 (0.0%)	1.000	0/1 (0.0%)	1.000	0/1 (0.0%)	1.000
*IQSEC2* (1)	0/1 (0.0%)	1.000	0/1 (0.0%)	1.000	0/1 (0.0%)	1.000
*KANSL1* (1)	1/1 (100.0%)	0.337	1/1 (100.0%)	0.301	1/1 (100.0%)	0.301
*KCNA1* (1)	0/1 (0.0%)	1.000	0/1 (0.0%)	1.000	0/1 (0.0%)	1.000
*KCNB1* (2)	2/2 (100.0%)	0.116	2/2 (100.0%)	0.093	2/2 (100.0%)	0.093
*KCNQ2* (6)	5/6 (83.3%)	0.022	5/6 (83.3%)	0.014	5/6 (83.3%)	0.014
*KCNT1* (3)	0/3 (0.0%)	0.548	0/3 (0.0%)	0.555	0/3 (33.3%)	0.555
*MECP2* (3)	0/3 (0.0%)	0.548	0/3 (0.0%)	0.555	0/3 (0.0%)	0.555
*SCN1A* (18)	14/18 (77.8%)	0.001	14/18 (77.8%)	<0.001	11/18 (61.1%)	0.014
*SCN2A* (3)	3/3 (100%)	0.041	3/3 (100.0%)	0.030	3/3 (100.0%)	0.030
*SCN3A* (1)	1/1 (100.0%)	0.337	1/1 (100.0%)	0.301	1/1 (100.0%)	0.301
*SCN8A* (3)	0/3 (0.0%)	0.548	0/3 (0.0%)	0.555	0/3 (0.0%)	0.555
*SLC6A1* (1)	1/1 (100.0%)	0.337	1/1 (100.0%)	0.301	1/1 (100.0%)	0.301
*SLC9A6* (2)	1/2 (50.0%)	1.000	1/2 (50.0%)	0.509	1/2 (50.0%)	0.509
*STXBP1* (4)	4/4 (100%)	0.015	4/4 (100%)	0.010	4/4 (100.0%)	0.010
*SYNGAP* (1)	0/1 (0.0%)	1.000	0/1 (0.0%)	1.000	0/1 (0.0%)	1.000
*WWOX* (1)	1/1 (100.0%)	0.337	1/1 (100.0%)	0.301	1/1 (100.0%)	0.301
*ZEB2* (1)	1/1 (100.0%)	0.337	1/1 (100.0%)	0.301	0/1 (0.0%)	1.000

### KD efficacy according to causative monogenic mutation

**Figure 2:**
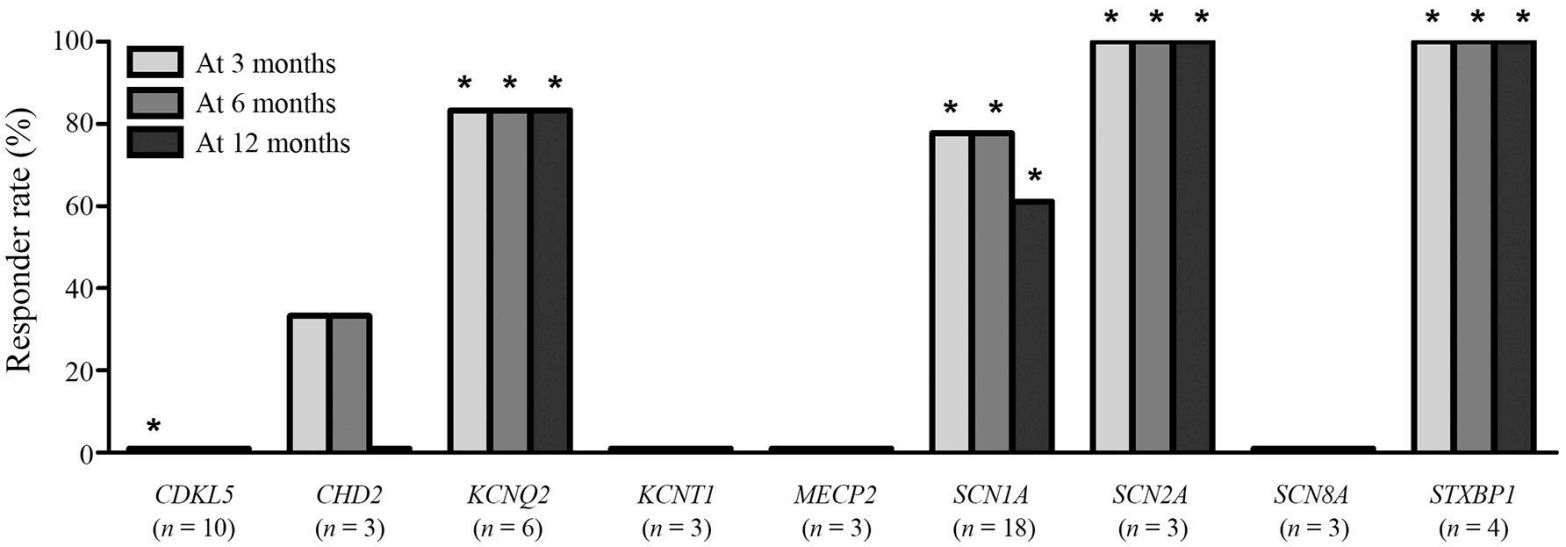
Responses to a ketogenic diet after 3, 6, and 12 months according to the identified pathogenic genetic mutations found in ≥3 patients. ^*^Responder rates that are significantly lower (*CDKL5*) or higher (the others) than the responder rate of patients without an identified genetic mutation.

The responder rates of patients with each pathogenic gene were compared with the responder rates of patients without identified genetic mutations at 3, 6, and 12 months after KD initiation (Table 3). At 3 months after KD implementation, patients with mutations in *KCNQ2* (*n* = 6, responder rate = 83.3%, *p* = 0.022), *SCN1A* (*n* = 18, responder rate = 77.8%, *p* = 0.001), *SCN2A* (*n* = 3, responder rate = 100.0%, *p* = 0.041), and *STXBP1* (*n* = 4, responder rate = 100.0%, *p* = 0.015) exhibited significantly better responses to KD than did patients without identified genetic mutations. In contrast, patients with mutations in *CDKL5* (*n* = 10, responder rate = 0.0%, *p* = 0.031) showed significantly poorer responses to KD than did patients with DEE without identified genetic mutations. At both 6 and 12 months after KD initiation, patients with mutations in *KCNQ2* (*n* = 6, responder rate = 83.3%, *p* = 0.014 and *p* = 0.014), *SCN1A* (*n* = 18, responder rate = 77.8 and 77.8%, *p* < 0.001 and *p* = 0.014), *SCN2A* (*n* = 3, responder rate = 100.0 and 100.0%, *p* = 0.030 and *p* = 0.030), and *STXBP1* (*n* = 4, responder rate = 100.0 and 100.0%, *p* = 0.010 and *p* = 0.010) still showed significantly better responses to KD compared to patients without identified genetic mutations. The responder rates to KD remained relatively consistent at 3, 6, and 12 months in all patients with pathogenic mutations (52.1, 49.3, and 43.8%) and patients with each genetic mutation, especially in *KCNQ2, SCN1A, SCN2A*, and *STXBP1* (Figure 2).

### KD efficacy according to causative mutations in epilepsy syndromes

As mentioned above, when patients were divided by syndromic diagnoses at the time of KD initiation, West syndrome was the most frequent, followed by Lennox-Gastaut syndrome, Dravet syndrome, and Ohtahara syndrome (Table 1). Among 67 West syndrome patients, pathogenic mutations were found in 20 (29.9%) patients, including 9 patients with CDKL5 mutations. Mutations in other genes were found in ≤ 2 patients for each gene. Responder rate of West syndrome patients with CDKL5 mutations was significantly lower than responder rate of West syndrome patients without CDKL5 mutations (0.0 vs. 41.4%, *p* = 0.021). Among 31 Lennox-Gastaut syndrome patients, 12 (38.7%) patients were identified with pathogenic mutations. Causative genes with mutations in Lennox-Gastaut syndrome patients were heterogeneous to analyze, with 3 boys with mutations in MECP2 gene and other 9 patients all identified with different genes from each other. All 18 Dravet syndrome patients were identified with mutations in SCN1A gene, and 14 (77.8%) patients showed a good response to KD at 3 months since initiation of KD. Of 17 Ohtahara syndrome patients, 15 (88.2%) patients were identified with causative mutations, including 6 KCNQ2 mutations, 3 STXBP1 mutations, and other mutations found in single patient each. Responder rate of Ohtahara syndrome patients with KCNQ2 or STXBP1 mutations showed a significantly better response to KD after 3 months compared to Ohtahara syndrome patients without KCNQ2 or STXBP1 mutations (88.9 vs. 37.5%, *p* = 0.043).

## Discussion

In our study, among the patients with DEE with various pathogenic mutations, patients with *SCN2A, STXBP1, KCNQ2*, and *SCN1A* mutations in particular showed better responses to KD, with responder rates of 100, 100, 83.3, and 77.8% respectively. However, patients with *CDKL5* mutations sho wed poorer responses to KD (responder rate = 0.0%) at 3 months after implementation. The responder rate to KD remained consistent at 3, 6, and 12 months after diet initiation when examined according to genotype.

The monogenic mutations that are responsible for DEE are increasingly being discovered with the advent of the NGS technique and targeted NGS or gene panel studies have been suggested as the most cost-effective genetic testing method for patients with DEE (14). Of the 333 patients in our study who underwent a DEE gene panel, 125 (37.5%) patients had an identified causative monogenic mutation; this incidence rate is similar to those reported in previous studies (15, 16), and such findings collectively support the usefulness of NGS panels in searching for the genetic causes of DEE. Moreover, our data provide useful insights regarding whom and when to recommend KD therapy for patients with gene-associated DEE.

To date, few reports on the efficacy of KD in patients with DEE with specific genotypes have been published (17–19), and of these, most are anecdotal reports or include short-term evaluations of the efficacy in only one specific genotype. In the present study, we assessed the long-term response to KD (over 12 months) in patients with each genotype of DEE. Additionally, we focused on patients who showed definite improvements after KD by strictly regarding only those patients who showed ≥90% seizure reduction as responders. Therefore, this study is significant because it is the first to assess the long-term efficacy of KD according to the various genetic causes of DEE. Moreover, we revealed the genotypes that are the most likely to show significant seizure reductions after KD.

The KD is reportedly effective for patients with DEE syndromes such as Dravet syndrome, West syndrome, Lennox-Gastaut syndrome, and epilepsy with myoclonic atonic seizures (7–10, 20–22). Efforts to identify phenotypes with clinical variables that can help predict which patients will show a better response to KD have been made (23). Our study showed that children with specific syndromes, especially those with an identified genetic cause, responded better to KD at 3 months than did those with syndromes without identified genetic causes. However, none of the other clinical variables we evaluated were related to a good KD response. In addition to the type of epileptic syndrome, we compared the efficacy of KD between different genotypes in order to reveal which genotypes are the most likely to respond well to KD. Our analysis demonstrated that patients with *SCN2A, STXBP1, KCNQ2*, and *SCN1A* mutations had more favorable responses to KD than did those without genetic mutations.

Previous studies have shown that patients with Dravet syndrome with *SCN1A* mutations respond well to KD (21, 24), as did a patient with an *SCN2A* mutation who was treated with a modified Atkins diet (19). Here, as well as in several other studies (25, 26), patients with *CDKL5* mutations showed poor responses to or only short-term efficacy of KD. Therefore, our findings regarding these genes are consistent with those of previous studies and confirm that patients with epileptic encephalopathy with *SCN1A* and *SCN2A* mutations respond well to KD, while patients with *CDKL5* mutations respond poorly to KD. Although prior studies showed that for patients with *STXBP1* encephalopathy, the response to KD was either slight or none (two case reports, each with one patient) (27, 28), in our study, all four patients with *STXBP1* mutations responded well to KD.

Among patients with West syndrome, patients with *CDKL5* mutations showed a significantly poorer response to KD than other West syndrome patients without *CDKL5* mutation. Among patients with Ohtahara syndrome, patients with *KCNQ2* or *STXBP1* mutations showed a significantly better response to KD than the others in this study. This is in line with the results from analysis by causative mutations in all DEE, showing that causative mutation is the important determining factor of response to KD as much as syndromic diagnoses in DEE. However, further study with larger sample size is warranted.

To our knowledge, no reports have evaluated the efficacy of KD in patients with *KCNQ2*-related neonatal epileptic encephalopathy. Here, 83.3% of patients with *KCNQ2* (5/6 patients) mutations responded to KD. However, none of our patients with *CHD2* (0/3), *KCNT1* (0/3), *SCN8A* (0/3), and *MECP2* (0/3, all boys) mutations showed a response to KD. Unfortunately, in patients with these genetic mutations, the KD efficacy was not statistically significant than was that in patients without identified genetic mutations owing to the small number of patients with each genetic mutation.

We understand the limitations of using an observational approach in this study and acknowledge that prospective or randomized controlled studies can provide stronger evidence regarding the efficacy of KD in patients with DEE with each pathogenic variant. We also acknowledge the very small sample size of this study, due to rare nature of DEE with identified pathogenic mutations. This study should therefore be regarded as elementary results demonstrating that specific genetic mutations show different response to KD. Due to the retrospective study design, the effects of KD on the developmental outcomes of these children were not evaluated. For patients without identified genetic mutations, some proportion of them is suspected to have a genetic origin owing to unknown pathogenic genes or genes that were not included in our NGS panel. Despite its limitations, the present study provides the first overview of the efficacy of KD in patients with DEE according to genotype and may serve as a basis for administering precision medicine.

## Conclusions

The KD was particularly effective in patients with DEE caused by *SCN2A, STXBP1, KCNQ2*, and *SCN1A* mutations and was not effective in patients with DEE caused by *CDKL5* mutations. These results will provide a rational basis for considering early, targeted KD treatment rather than the traditional trial and error approach to epilepsy therapy in this group of patients. Furthermore, our data may help avoid overtreatment with KD in patients with DEE with *CDKL5* mutations.

## Author contributions

DJ and HK conceptualized and designed the study, reviewed and revised the manuscript. DJ and AK carried out the initial analyses, drafted the initial manuscript. SL and JC carried out the initial analyses. SK, H-CK, and JL collected data and critically reviewed the manuscript.

### Conflict of interest statement

The authors declare that the research was conducted in the absence of any commercial or financial relationships that could be construed as a potential conflict of interest.

## Abbreviations

AED: antiepileptic drug
DEE: developmental and epileptic encephalopathy
KD: ketogenic diet
NGS: next-generation sequencing.
