# Supplementary material for: The Efficacy of Ketogenic Diet for Specific Genetic Mutation in Developmental and Epileptic Encephalopathy
Source: Front Neurol. 2018 Jul 16;9:530. doi: 10.3389/fneur.2018.00530 (PMC6054992; doi:10.3389/fneur.2018.00530)
Supplement: Supplementary file 1 [file Table_1.DOCX]

**Table S1** List of 172 Targeted Genes Included in the Developmental and Epileptic Encephalopathy Panel

| Gene | OMIM | Full Name | Cytogenetic Location |
| --- | --- | --- | --- |
| *AARS* | 601065 | ALANYL-tRNA SYNTHETASE | 16q22.1 |
| *ABAT* | 137150 | 4-AMINOBUTYRATE AMINOTRANSFERASE | 16p13.2 |
| *ACADL* | 609576 | ACYL-CoA DEHYDROGENASE, LONG-CHAIN | 2q34 |
| *ACADM* | 607008 | ACYL-CoA DEHYDROGENASE, MEDIUM-CHAIN | 1p31.1 |
| *ACADS* | 606885 | ACYL-CoA DEHYDROGENASE, SHORT-CHAIN | 12q24.31 |
| *ACY1* | 104620 | AMINOACYLASE 1 | 3p21.2 |
| *ADGRV1* | 602851 | ADHESION G PROTEIN-COUPLED RECEPTOR V1 | 5q14.3 |
| *ADSL* | 608222 | ADENYLOSUCCINATE LYASE | 22q13.1 |
| *ALAD* | 125270 | DELTA-AMINOLEVULINATE DEHYDRATASE | 9q32 |
| *ALAS2* | 301300 | DELTA-AMINOLEVULINATE SYNTHASE 2 | Xp11.21 |
| *ALDH4A1* | 606811 | ALDEHYDE DEHYDROGENASE, FAMILY 4, SUBFAMILY A, MEMBER 1 | 1p36.13 |
| *ALDH7A1* | 107323 | ALDEHYDE DEHYDROGENASE 7 FAMILY, MEMBER A1 | 5q23.2 |
| *ALG13* | 300776 | ASPARAGINE-LINKED GLYCOSYLATION 13 | Xq23 |
| *ALPL* | 171760 | ALKALINE PHOSPHATASE, LIVER | 1p36.12 |
| *AMT* | 238310 | AMINOMETHYLTRANSFERASE | 3p21.31 |
| *ARHGEF15* | 608504 | RHO GUANINE NUCLEOTIDE EXCHANGE FACTOR 15 | 17p13.1 |
| *ARHGEF9* | 300429 | RHO GUANINE NUCLEOTIDE EXCHANGE FACTOR 9 | Xq11.1 |
| *ARX* | 300382 | ARISTALESS-RELATED HOMEOBOX, X-LINKED | Xp21.3 |
| *ASNS* | 108370 | ASPARAGINE SYNTHETASE | 7q21.3 |
| *ASPM* | 605481 | ABNORMAL SPINDLE-LIKE, MICROCEPHALY-ASSOCIATED | 1q31.3 |
| *ATP13A2* | 610513 | ATPase, TYPE 13A2 | 1p36.13 |
| *ATP6AP2* | 300556 | ATPase, H+ TRANSPORTING, LYSOSOMAL, ACCESSORY PROTEIN 2 | Xp11.4 |
| *BRAT1* | 614506 | BRCA1-ASSOCIATED ATM ACTIVATOR 1 | 7p22.3 |
| *BTD* | 609019 | BIOTINIDASE | 3p25.1 |
| *CACNA1A* | 601011 | CALCIUM CHANNEL, VOLTAGE-DEPENDENT, P/Q TYPE, ALPHA-1A SUBUNIT | 19p13.13 |
| *CACNB4* | 601949 | CALCIUM CHANNEL, VOLTAGE-DEPENDENT, BETA-4 SUBUNIT | 2q23.3 |
| *CASK* | 300172 | CALCIUM/CALMODULIN-DEPENDENT SERINE PROTEIN KINASE | Xp11.4 |
| *CASR* | 601199 | CALCIUM-SENSING RECEPTOR | 3q13.3-q21.1 |
| *CBS* | 613381 | CYSTATHIONINE BETA-SYNTHASE | 21q22.3 |
| *CDKL5* | 300203 | CYCLIN-DEPENDENT KINASE-LIKE 5 | Xp22.13 |
| *CHD2* | 602119 | CHROMODOMAIN HELICASE DNA-BINDING PROTEIN 2 | 15q26.1 |
| *CHRNA2* | 118502 | CHOLINERGIC RECEPTOR, NEURONAL NICOTINIC, ALPHA POLYPEPTIDE 2 | 8p21.2 |
| *CHRNA4* | 118504 | CHOLINERGIC RECEPTOR, NEURONAL NICOTINIC, ALPHA POLYPEPTIDE 4 | 20q13.33 |
| *CHRNA7* | 118511 | CHOLINERGIC RECEPTOR, NEURONAL NICOTINIC, ALPHA POLYPEPTIDE 7 | 15q13.3 |
| *CHRNB2* | 118507 | CHOLINERGIC RECEPTOR, NEURONAL NICOTINIC, BETA POLYPEPTIDE 2 | 1q21.3 |
| *CLCN4* | 302910 | CHLORIDE CHANNEL 4 | Xp22.2 |
| *CLN3* | 607042 | CLN3 GENE | 16p12.1 |
| *CLN5* | 608102 | CLN5 GENE | 13q22.3 |
| *CLN6* | 606725 | CLN6 GENE | 15q23 |
| *CLN8* | 607837 | CLN8 GENE | 8p23.3 |
| *CNTNAP2* | 604569 | CONTACTIN-ASSOCIATED PROTEIN-LIKE 2 | 7q35-q36 |
| *COL4A1* | 120130 | COLLAGEN, TYPE IV, ALPHA-1 | 13q34 |
| *CPOX* | 612732 | COPROPORPHYRINOGEN OXIDASE | 3q11.2 |
| *CPT1A* | 600528 | CARNITINE PALMITOYLTRANSFERASE I, LIVER | 11q13.3 |
| *CPT1B* | 601987 | CARNITINE PALMITOYLTRANSFERASE I, MUSCLE | 22q13.33 |
| *CPT2* | 600650 | CARNITINE PALMITOYLTRANSFERASE II | 1p32.3 |
| *CSTB* | 601145 | CYSTATIN B | 21q22.3 |
| *CTSD* | 116840 | CATHEPSIN D | 11p15.5 |
| *CTSF* | 603539 | CATHEPSIN F | 11q13.2 |
| *DNAJC5* | 611203 | DNAJ/HSP40 HOMOLOG, SUBFAMILY C, MEMBER 5 | 20q13.33 |
| *DNM1* | 602377 | DYNAMIN 1 | 9q34.11 |
| *DOCK7* | 615730 | DEDICATOR OF CYTOKINESIS 7 | 1p31.3 |
| *DYRK1A* | 600855 | DUAL-SPECIFICITY TYROSINE PHOSPHORYLATION-REGULATED KINASE 1A | 21q22.13 |
| *EEF1A2* | 602959 | EUKARYOTIC TRANSLATION ELONGATION FACTOR 1, ALPHA-2 | 20q13.33 |
| *EPM2A* | 607566 | EPM2A GENE | 6q24.3 |
| *FARS2* | 611592 | PHENYLALANYL-tRNA SYNTHETASE 2, MITOCHONDRIAL | 6p25.1 |
| *FECH* | 612386 | FERROCHELATASE | 18q21.31 |
| *FOLR1* | 136430 | FOLATE RECEPTOR 1, ADULT | 11q13.4 |
| *FOXG1* | 164874 | FORKHEAD BOX G1 | 14q12 |
| *GABBR2* | 607340 | GAMMA-AMINOBUTYRIC ACID B RECEPTOR 2 | 9q22.33 |
| *GABRA1* | 137160 | GAMMA-AMINOBUTYRIC ACID RECEPTOR, ALPHA-1 | 5q34 |
| *GABRB3* | 137192 | GAMMA-AMINOBUTYRIC ACID RECEPTOR, BETA-3 | 15q12 |
| *GABRG2* | 137164 | GAMMA-AMINOBUTYRIC ACID RECEPTOR, GAMMA-2 | 5q34 |
| *GAMT* | 601240 | GUANIDINOACETATE METHYLTRANSFERASE | 19p13.3 |
| *GATM* | 602360 | L-ARGININE:GLYCINE AMIDINOTRANSFERASE | 15q21.1 |
| *GCSH* | 238330 | GLYCINE CLEAVAGE SYSTEM H PROTEIN | 16q23.2 |
| *GLDC* | 238300 | GLYCINE DECARBOXYLASE | 9p24.1 |
| *GNAO1* | 139311 | GUANINE NUCLEOTIDE-BINDING PROTEIN, ALPHA-ACTIVATING ACTIVITY POLYPEPTIDE O | 16q13 |
| *GOSR2* | 604027 | GOLGI SNAP RECEPTOR COMPLEX MEMBER 2 | 17q21.32 |
| *GRIN1* | 138249 | GLUTAMATE RECEPTOR, IONOTROPIC, N-METHYL-D-ASPARTATE, SUBUNIT 1 | 9q34.3 |
| *GRIN2A* | 138253 | GLUTAMATE RECEPTOR, IONOTROPIC, N-METHYL-D-ASPARTATE, SUBUNIT 2A | 16p13.2 |
| *GRIN2B* | 138252 | GLUTAMATE RECEPTOR, IONOTROPIC, N-METHYL-D-ASPARTATE, SUBUNIT 2B | 12p13.1 |
| *GRN* | 138945 | GRANULIN PRECURSOR | 17q21.31 |
| *HADH* | 601609 | 3-HYDROXYACYL-CoA DEHYDROGENASE | 4q25 |
| *HADHA* | 600890 | HYDROXYACYL-CoA DEHYDROGENASE/3-KETOACYL-CoA THIOLASE/ENOYL-CoA HYDRATASE, ALPHA SUBUNIT | 2p23.3 |
| *HCN1* | 602780 | HYPERPOLARIZATION-ACTIVATED CYCLIC NUCLEOTIDE-GATED POTASSIUM CHANNEL 1 | 5p12 |
| *HCN4* | 605206 | HYPERPOLARIZATION-ACTIVATED CYCLIC NUCLEOTIDE-GATED POTASSIUM CHANNEL 4 | 15q24.1 |
| *HFE* | 613609 | HFE GENE | 6p22.2 |
| *HLCS* | 609018 | HOLOCARBOXYLASE SYNTHETASE | 21q22.13 |
| *HMBS* | 609806 | HYDROXYMETHYLBILANE SYNTHASE | 11q23.3 |
| *HNRNPU* | 602869 | HETEROGENEOUS NUCLEAR RIBONUCLEOPROTEIN U | 1q44 |
| *IQSEC2* | 300522 | IQ MOTIF- AND SEC7 DOMAIN-CONTAINING PROTEIN 2 | Xp11.22 |
| *KANSL1* | 612452 | KAT8 REGULATORY NSL COMPLEX, SUBUNIT 1 | 17q21.31 |
| *KCNA1* | 176260 | POTASSIUM CHANNEL, VOLTAGE-GATED, SHAKER-RELATED SUBFAMILY, MEMBER 1 | 12p13.32 |
| *KCNA2* | 176262 | POTASSIUM CHANNEL, VOLTAGE-GATED, SHAKER-RELATED SUBFAMILY, MEMBER 2 | 1p13.3 |
| *KCNB1* | 600397 | POTASSIUM CHANNEL, VOLTAGE-GATED, SHAB-RELATED SUBFAMILY, MEMBER 1 | 20q13.13 |
| *KCNC1* | 176258 | POTASSIUM CHANNEL, VOLTAGE-GATED, SHAW-RELATED SUBFAMILY, MEMBER 1 | 11p15.1 |
| *KCNH5* | 605716 | POTASSIUM CHANNEL, VOLTAGE-GATED, SUBFAMILY H, MEMBER 5 | 14q23.2 |
| *KCNJ10* | 602208 | POTASSIUM CHANNEL, INWARDLY RECTIFYING, SUBFAMILY J, MEMBER 10 | 1q23.2 |
| *KCNJ11* | 600937 | POTASSIUM CHANNEL, INWARDLY RECTIFYING, SUBFAMILY J, MEMBER 11 | 11p15.1 |
| *KCNMA1* | 600150 | POTASSIUM CHANNEL, CALCIUM-ACTIVATED, LARGE CONDUCTANCE, SUBFAMILY M, ALPHA MEMBER 1 | 10q22.3 |
| *KCNQ2* | 602235 | POTASSIUM CHANNEL, VOLTAGE-GATED, KQT-LIKE SUBFAMILY, MEMBER 2 | 20q13.33 |
| *KCNQ3* | 602232 | POTASSIUM CHANNEL, VOLTAGE-GATED, KQT-LIKE SUBFAMILY, MEMBER 3 | 8q24.22 |
| *KCNT1* | 608167 | POTASSIUM CHANNEL, SUBFAMILY T, MEMBER 1 | 9q34.3 |
| *KCTD7* | 611725 | POTASSIUM CHANNEL TETRAMERIZATION DOMAIN-CONTAINING PROTEIN 7 | 7q11.21 |
| *KPNA7* | 614107 | KARYOPHERIN ALPHA-7 | 7q22.1 |
| *LGI1* | 604619 | LEUCINE-RICH GENE, GLIOMA-INACTIVATED, 1 | 10q23.33 |
| *LIAS* | 607031 | LIPOIC ACID SYNTHASE | 4p14 |
| *MAGI2* | 606382 | MEMBRANE-ASSOCIATED GUANYLATE KINASE, WW AND PDZ DOMAINS-CONTAINING, 2 | 7q21.11 |
| *MBD5* | 611472 | METHYL-CpG-BINDING DOMAIN PROTEIN 5 | 2q23.1 |
| *MECP2* | 300005 | METHYL-CpG-BINDING PROTEIN 2 | Xq28 |
| *MEF2C* | 600662 | MADS BOX TRANSCRIPTION ENHANCER FACTOR 2, POLYPEPTIDE C | 5q14.3 |
| *MFSD8* | 611124 | MAJOR FACILITATOR SUPERFAMILY DOMAIN-CONTAINING PROTEIN 8 | 4q28.2 |
| *MMADHC* | 611935 | MMADHC GENE | 2q23.2 |
| *MTHFR* | 607093 | 5,10-METHYLENETETRAHYDROFOLATE REDUCTASE | 1p36.22 |
| *MTR* | 156570 | 5-METHYLTETRAHYDROFOLATE-HOMOCYSTEINE S-METHYLTRANSFERASE | 1q43 |
| *MTRR* | 602568 | METHIONINE SYNTHASE REDUCTASE | 5p15.31 |
| *NECAP1* | 611623 | NECAP ENDOCYTOSIS-ASSOCIATED PROTEIN 1 | 12p13.31 |
| *NHLRC1* | 608072 | NHL REPEAT-CONTAINING 1 GENE | 6p22.3 |
| *NRXN1* | 600565 | NEUREXIN I | 2p16.3 |
| *OPHN1* | 300127 | OLIGOPHRENIN 1 | Xq12 |
| *PAH* | 612349 | PHENYLALANINE HYDROXYLASE | 12q23.2 |
| *PC* | 608786 | PYRUVATE CARBOXYLASE | 11q13.2 |
| *PCDH19* | 300460 | PROTOCADHERIN 19 | Xq22.1 |
| *PHGDH* | 606879 | PHOSPHOGLYCERATE DEHYDROGENASE | 1p12 |
| *PIGA* | 311770 | PHOSPHATIDYLINOSITOL GLYCAN ANCHOR BIOSYNTHESIS CLASS A PROTEIN | Xp22.2 |
| *PIGQ* | 605754 | PHOSPHATIDYLINOSITOL GLYCAN ANCHOR BIOSYNTHESIS CLASS Q PROTEIN | 16p13.3 |
| *PLCB1* | 607120 | PHOSPHOLIPASE C, BETA-1 | 20p12.3 |
| *PNKP* | 605610 | POLYNUCLEOTIDE KINASE 3-PRIME PHOSPHATASE | 19q13.33 |
| *PNPO* | 603287 | PYRIDOXAMINE 5-PRIME-PHOSPHATE OXIDASE | 17q21.32 |
| *POLG* | 174763 | POLYMERASE, DNA, GAMMA | 15q26.1 |
| *PPOX* | 600923 | PROTOPORPHYRINOGEN OXIDASE | 1q23.3 |
| *PPT1* | 600722 | PALMITOYL-PROTEIN THIOESTERASE 1 | 1p34.2 |
| *PRICKLE1* | 608500 | PRICKLE, DROSOPHILA, HOMOLOG OF, 1 | 12q12 |
| *PRICKLE2* | 608501 | PRICKLE, DROSOPHILA, HOMOLOG OF, 2 | 3p14.1 |
| *PRODH* | 606810 | PROLINE DEHYDROGENASE (OXIDASE) 1 | 22q11.21 |
| *PRRT2* | 614386 | PROLINE-RICH TRANSMEMBRANE PROTEIN 2 | 16p11.2 |
| *PURA* | 600473 | PURINE-RICH ELEMENT-BINDING PROTEIN A | 5q31.3 |
| *QARS* | 603727 | GLUTAMINYL-tRNA SYNTHETASE | 3p21.31 |
| *SCARB2* | 602257 | SCAVENGER RECEPTOR CLASS B, MEMBER 2 | 4q21.1 |
| *SCN1A* | 182389 | SODIUM CHANNEL, NEURONAL TYPE I, ALPHA SUBUNIT | 2q24.3 |
| *SCN1B* | 600235 | SODIUM CHANNEL, VOLTAGE-GATED, TYPE I, BETA SUBUNIT | 19q13.11 |
| *SCN2A* | 182390 | SODIUM CHANNEL, VOLTAGE-GATED, TYPE II, ALPHA SUBUNIT | 2q24.3 |
| *SCN3A* | 182391 | SODIUM CHANNEL, VOLTAGE-GATED, TYPE III, ALPHA SUBUNIT | 2q24.3 |
| *SCN8A* | 600702 | SODIUM CHANNEL, VOLTAGE-GATED, TYPE VIII, ALPHA SUBUNIT | 12q13.13 |
| *SCN9A* | 603415 | SODIUM CHANNEL, VOLTAGE-GATED, TYPE IX, ALPHA SUBUNIT | 2q24.3 |
| *SETBP1* | 611060 | SET-BINDING PROTEIN 1 | 18q12.3 |
| *SIK1* | 605705 | SALT-INDUCIBLE KINASE 1 | 21q22.3 |
| *SLC13A5* | 608305 | SOLUTE CARRIER FAMILY 13 (SODIUM-DEPENDENT CITRATE TRANSPORTER), MEMBER 5 | 17p13.1 |
| *SLC19A3* | 606152 | SOLUTE CARRIER FAMILY 19 (THIAMINE TRANSPORTER), MEMBER 3 | 2q36.3 |
| *SLC22A5* | 603377 | SOLUTE CARRIER FAMILY 22 (ORGANIC CATION TRANSPORTER), MEMBER 5 | 5q31.1 |
| *SLC25A20* | 613698 | SOLUTE CARRIER FAMILY 25 (CARNITINE/ACYLCARNITINE TRANSLOCASE), MEMBER 20 | 3p21.31 |
| *SLC25A22* | 609302 | SOLUTE CARRIER FAMILY 25 (MITOCHONDRIAL CARRIER, GLUTAMATE), MEMBER 22 | 11p15.5 |
| *SLC25A29* | 615064 | SOLUTE CARRIER FAMILY 25 (CARNITINE/ACYLCARNITINE TRANSLOCASE), MEMBER 29 | 14q32.2 |
| *SLC2A1* | 138140 | SOLUTE CARRIER FAMILY 2 (FACILITATED GLUCOSE TRANSPORTER), MEMBER 1 | 1p34.2 |
| *SLC46A1* | 611672 | SOLUTE CARRIER FAMILY 46 (FOLATE TRANSPORTER), MEMBER 1 | 17q11.2 |
| *SLC6A1* | 137165 | SOLUTE CARRIER FAMILY 6 (NEUROTRANSMITTER TRANSPORTER, GABA), MEMBER 1 | 3p25.3 |
| *SLC6A8* | 300036 | SOLUTE CARRIER FAMILY 6 (NEUROTRANSMITTER TRANSPORTER, CREATINE), MEMBER 8 | Xq28 |
| *SLC9A6* | 300231 | SOLUTE CARRIER FAMILY 9, MEMBER 6 | Xq26.3 |
| *SMARCA2* | 600014 | SWI/SNF-RELATED, MATRIX-ASSOCIATED, ACTIN-DEPENDENT REGULATOR OF CHROMATIN, SUBFAMILY A, MEMBER 2 | 9p24.3 |
| *SPTAN1* | 182810 | SPECTRIN, ALPHA, NONERYTHROCYTIC 1 | 9q34.11 |
| *SRPX2* | 300642 | SUSHI REPEAT-CONTAINING PROTEIN, X-LINKED, 2 | Xq22.1 |
| *ST3GAL3* | 606494 | ST3 BETA-GALACTOSIDE ALPHA-2,3-SIALYLTRANSFERASE 3 | 1p34.1 |
| *ST3GAL5* | 604402 | ST3 BETA-GALACTOSIDE ALPHA-2,3-SIALYLTRANSFERASE 5 | 2p11.2 |
| *STX1B* | 601485 | SYNTAXIN 1B | 16p11.2 |
| *STXBP1* | 602926 | SYNTAXIN-BINDING PROTEIN 1 | 9q34.11 |
| *SYN1* | 313440 | SYNAPSIN I | Xp11.3-p11.2 |
| *SYNGAP1* | 603384 | SYNAPTIC RAS-GTPase-ACTIVATING PROTEIN 1 | 6p21.32 |
| *SZT2* | 615463 | SEIZURE THRESHOLD 2, MOUSE, HOMOLOG OF | 1p34.2 |
| *TBC1D24* | 613577 | TBC1 DOMAIN FAMILY, MEMBER 24 | 16p13.3 |
| *TBL1XR1* | 608628 | TRANSDUCIN-BETA-LIKE 1 RECEPTOR 1 | 3q26.32 |
| *TCF4* | 602272 | TRANSCRIPTION FACTOR 4 | 18q21.2 |
| *TNK2* | 606994 | TYROSINE KINASE, NONRECEPTOR, 2 | 3q29 |
| *TPP1* | 607998 | TRIPEPTIDYL PEPTIDASE I | 11p15.4 |
| *TSEN54* | 608755 | tRNA SPLICING ENDONUCLEASE 54, S. CEREVISIAE, HOMOLOG OF | 17q25.1 |
| *UBE2A* | 312180 | UBIQUITIN-CONJUGATING ENZYME E2A | Xq24 |
| *UBE3A* | 601623 | UBIQUITIN-PROTEIN LIGASE E3A | 15q11.2 |
| *UROD* | 613521 | UROPORPHYRINOGEN DECARBOXYLASE | 1p34.1 |
| *UROS* | 606938 | UROPORPHYRINOGEN III SYNTHASE | 10q26.2 |
| *WDR62* | 613583 | WD REPEAT-CONTAINING PROTEIN 62 | 19q13.12 |
| *WWOX* | 605131 | WW DOMAIN-CONTAINING OXIDOREDUCTASE | 16q23.1-q23.2 |
| *ZEB2* | 605802 | ZINC FINGER E BOX-BINDING HOMEOBOX 2 | 2q22.3 |

OMIM, online mendelian inheritance in man.
